# The Philosophy and Art of Happiness

**Published:** 1855-01

**Authors:** 


### THE PHILOSOPHY AND ART OF HAPPINESS.

The glowing golden tint that colors the distant western hill, as the sun behind it is sinking into evening, recedes before the approaching traveler, as swiftly as his own gaunt shadow pursues; and far beyond gilds a country he can never reach before its brightness be departed. So human happiness is an ideal delight pictured before the mind and heart, which men seek to attain, but never reach.

All that is called happiness is in reality but the pursuit of it. Men are said to be in search of health, when they go roaming over hills to exercise their limbs, and breathe the fresh, pure air. At the end of their journeys, they are known to have found nothing, except that the search was the only treasure they sought. In the same manner happiness is the exhilarating effect upon man of every thing he pursues in order to render him happy.

All minds are differently constituted. Therefore, the same object must make upon each a correspondingly different impression; also, to produce like impressions of an object in several minds, the object must be in a slight degree differently conceived by each. One thing differently presented to correspondingly dissimilar minds, may cause like impressions in all. Moreover, since every mind at different times is often in different states, an object brought before it will not make always a like impression. In order, then, that the experience of a person, derived from a particular object, may remain unaltered for a length of time, those things which occasion experiences must be varied during that time, to accord with the changing phases of the mind.

For example, to produce in a person the emotion of happiness for any period, there must be presented to his mind, at every interval during that period, the precise state of things which, in every successive mental condition, will occasion the experience.

It is obvious, upon the mere recognition of these truths, that in causing an experience of happiness, or of any other emotion or state, a thousand times more depends upon the quality and condition of the mind, than upon the things which operate upon its faculties.

It therefore becomes a useful duty of man to discover and practice a discipline by which he may educate his mind to conceive everything in such a manner as will produce in him a continued pleasant experience; for over the external chances and accidents of life, upon which his happiness in part depends, he has only a weak control; but he has power to cause his mind, when his aim is the pursuit of happiness, to create and retain only such experiences as will make him happy.

An important inquiry immediately arises: what particular condition of the mind is conducive to the highest happiness? The answer to this question is brief, and the reason simple. The mind that is ripened in the Christian faith, and humbled yet exalted in presence of its divine Author and Finisher, whom it worships in love, is the only one that has *capacity* to experience the completest happiness that belongs to earth. Still more is such a mind the only one that is able to achieve from ill-boding circumstances, that would make worldly men wretched, a sweet serenity of joy, which though born on earth may yet be redolent of heaven. The man of the world, though he be conscious that happiness is a child of the mind, still neither expects, nor seeks, to be happy beyond the degree which he attains through the mind itself, unaided by any strength beside its own. Therefore, though amid common gloomy scenes of life he may be contented, he must in a crisis of adversity be overwhelmed. His unassisted mind, while of its own vigor it can accomplish wonders of endurance, beyond a limited strength is powerless to battle off the despair that follows disaster. He may watch his heroism tremble, and behold his spirit fail and sink. The reverses of fortune may transpire so suddenly and be so blighting, as to render him a misanthrope, who will contemplate even his existence with bitterness.

But the true Christian is never so stricken down. If he be wise and thoughtful, he knows that to make his happiness independent of the uncertainties of the world, his mind needs a sustaining strength superadded to its own; he asks of the "Giver of every good and perfect gift" that there may be imparted to it a divine energy which shall enable it to endure to the end. Or, if he be a plain man, ignorant of the cause of his weakness and conscious only of the fact, he instinctively seeks aid from Heaven; and if he have faith in the fulfilment of the promises of God, he is fortified by Omnipotence against the world.

In this manner the happiness of the true Christian is always unsubjected to the chance of things. Whether he bear the burden which the world calls sorrow, or is crowned with the garlands it calls joy, he is still happy. "Be of *good cheer*," said the Saviour to his disciples; "for ye have *overcome* the world." But the mind that refuses the Spirit of God, though it be happy while fortune is propitious, sinks into gloom when reverses come; and, having nothing to which it may cling when hope is faint and strength is weary, pines in despair. This is not the fate of those only who are destitute of moral principle. Men, high-minded and learned, have sought to achieve happiness by the power of *virtue*, and miserably failed; *religion only* is ever triumphant.

In the light of the philosophy here delineated, the reason is clear that the Christian can say with boldness and truth that even suffering is a touchstone of happiness. Suffering is a law of life, ordained by the Deity, and universally illustrated in the history of mankind; if it were not, we would never grow tired of pleasure, but now we are wearied by the most coveted gratifications. The aim of life is the pursuit of happiness; this has been appointed by the Creator. Therefore, it must appear, even to the dim eye of human logic, a plain truth that men may be happy while they suffer.

To say in naked words that suffering produces happiness; that anguish, intense and lasting, may yield a joy, exquisite and ineffable, would perhaps appear to the world an absurdity meriting its ridicule. It is, nevertheless, a truth; and one which ranks among the highest that man can comprehend. It is denied only because it is, to those who reject it, an unexplained mystery. There are legions of heavenly experiences that cannot be made plain to one man from mere recital of their phenomena by another; they must be felt in order to be comprehended. St. Paul declares, "When I am weak, then am I strong;" and the Saviour said, "He that humbleth himself shall be exalted." The letter of these declarations may seem paradoxical, but their spirit is clear and true. Like them is this, that suffering is an almoner of happiness. To those who have tasted the cup of bitterness, and rejoiced while they drank the gall, it is neither meaningless nor false. Unto such there is no mystery of meaning in the "joy of grief." They know it is even sweeter than the joy of pleasure. If among the learned in the wisdom of this world, none can interpret the full significance of this saying, many a mother, who has planted violets over the grave of her infant, while she has gone sorrowing that it died, has yet rejoiced with rapturous delight that it became an angel of Heaven. That worldly men are ignorant of this wonderful wisdom is not strange to the Christian philosopher. Christ himself said, "I thank thee, O Father, that thou hast *hid* these things from the *wise and prudent*, and hast revealed them unto babes."

It is a mystic wonder to men of mere cold morality, that while they never will deny that happiness is the aim of living, they know that trials and griefs are the most frequent incidents of life. They have yet to learn that the crown of happiness is interwoven of prosperity and adversity, and that what they call the unlovely leaves, are often the most numerous in the garland.

Happiness, then, is not a chance that distils a rare blessing upon the mind and heart, as the morning breathes its dew upon the flowers. Tradition relates that of the most wonderful arts of the ancient orientals, one was to mould a wine-cup of delicate porcelain so cunningly, that when empty it would appear pure and plain, but which a draught of pleasant wine, or treacherous poison, would illumine with pictures, brilliant and radiant with beauty. In like manner, the most wonderful art of life—the art of being happy—is so to refine the rarer material of the mind, that whether it be filled, as a goblet, with thoughts of sweet or bitter things, it shall still ever be radiant with experiences of glowing, heavenly joy.—*N. Y. Observer.*

